# The new exploration of pure total flavonoids extracted from *Citrus maxima (Burm.) Merr.* as a new therapeutic agent to bring health benefits for people

**DOI:** 10.3389/fnut.2022.958329

**Published:** 2022-10-06

**Authors:** Shuning Ding, Peipei Wang, Xi Pang, Leyin Zhang, Lihui Qian, Xinru Jia, Wenqian Chen, Shanming Ruan, Leitao Sun

**Affiliations:** ^1^The First School of Clinical Medicine, Zhejiang Chinese Medical University, Hangzhou, China; ^2^Department of Medical Oncology, Hangzhou TCM Hospital of Zhejiang Chinese Medical University (Hangzhou Hospital of Traditional Chinese Medicine), Hangzhou, China; ^3^Department of Medical Oncology, The First Affiliated Hospital of Zhejiang Chinese Medical University (Zhejiang Provincial Hospital of Traditional Chinese Medicine), Hangzhou, China

**Keywords:** flavonoids, extraction, metabolism, gut microbiota, *Citrus maxima (Burm.) merr.*

## Abstract

The peel and fruit of *Citrus* varieties have been a raw material for some traditional Chinese medicine (TCM). Pure total flavonoids from *Citrus maxima (Burm.) Merr*. (PTFC), including naringin, hesperidin, narirutin, and neohesperidin, have been attracted increasing attention for their multiple clinical efficacies. Based on existing *in vitro* and *in vivo* research, this study systematically reviewed the biological functions of PTFC and its components in preventing or treating liver metabolic diseases, cardiovascular diseases, intestinal barrier dysfunction, as well as malignancies. PTFC and its components are capable of regulating glycolipid metabolism, blocking peroxidation and persistent inflammation, inhibiting tumor progression, protecting the integrity of intestinal barrier and positively regulating intestinal microbiota, while the differences in fruit cultivation system, picking standard, manufacturing methods, delivery system and individual intestinal microecology will have impact on the specific therapeutic effect. Thus, PTFC is a promising drug for the treatment of some chronic diseases, as well as continuous elaborate investigations are necessary to improve its effectiveness and bioavailability.

## Introduction

*Citrus maxima (Burm.) Merr.* (Changshanhuyou Tangelo) belongs to the genus *Citrus* of the *Rutaceae* family. It is a locally characteristic *Citrus* species planted in Changshan County, Zhejiang Province, China. Moreover, it refers to a hybrid between pomelo and sweet orange conducted by natural process and human intervention, which is considered a novel species retaining all the abilities of its “parents.” Geographical conditions (e.g., the humid monsoon climate and fertile red soil) contribute to the unique planting. Accordingly, *Citrus* varieties have been recognized as pillars of local agriculture with the largest output value in southeastern China. As the explorations on *C. maxima (Burm.) Merr.* have been deepened, its clinical efficacy in reducing accumulation and promoting Qi has been increasingly recognized, and it was officially accepted as a traditional Chinese medicine (TCM) in 2015. It is bitter, pungent, and sour with slight cold nature, as well as enter into spleen and stomach meridians. During the processing of *C. maxima (Burm.) Merr*., most of the pulp is removed for consumption, and the peel is fried to deep yellow, which acts as the valuable part enriching bioactive ingredients. The remaining parts of peel that are damaged, worm-eaten or wasted can be burned and used as soil fertilizer to nourish fruit trees.

**FIGURE 1:**
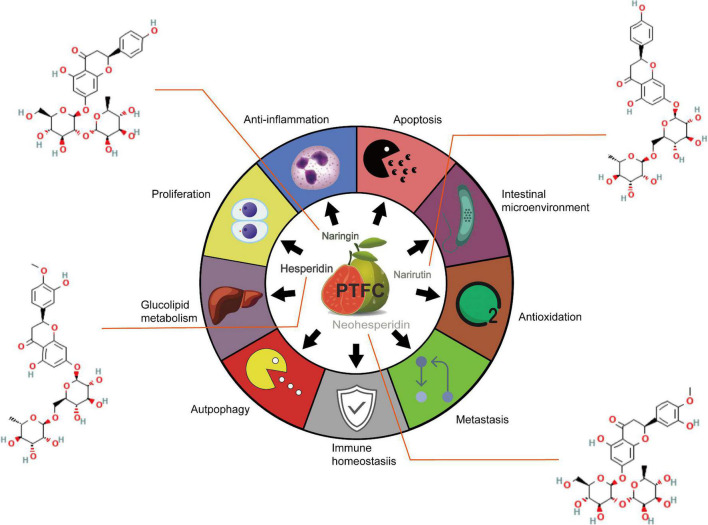
Summary of the therapeutic potentials of PTFC administration on hepatic metabolic disorder, cardiovascular events, malignancies development and intestinal barrier dysfunction.

The comprehensive development of *Citrus* species leads to an increase in economic income while triggering the transformation and upgrading of the traditional agricultural product. The rapid development of *Citrus* peel is primarily dependent on its massive availability and cost-effectiveness. Accumulating evidence suggests that there are abundant flavonoids with multiple beneficial effects in the peel of *Citrus* varieties (1). Epidemiological evidence suggests that the intake of food with high-level flavonoids and low-level fatty acids (e.g., fruit and vegetables) is directly correlated with a decreased risk of human diseases [e.g., cardiovascular diseases (CVD), diabetes, and cancers] (2–4). In addition, existing research has suggested that flavonoids play a vital role in the aging process by providing antioxidants and avoiding a vicious cycle of oxidative stress, tissue damage, and inflammatory processes (5). Nevertheless, the above effects have been controversial due to the individualized differences in the absorption and metabolism caused by the varied transformation of gut microbiota (6). A previous study has indicated that the urinary excretion level of flavonoid metabolism in healthy volunteers compared with the absorbed flavonoids reaches 45.9, 64.2, and 100%, respectively, implying that individualized differences in gut microbiota directly affects the bioavailability (7, 8). The result of high-performance liquid chromatography (HPLC) indicated that four pure flavonoids compounds exerted from *Citrus maxima (Burm.) Merr*. (PTFC) were found, including naringin, neohesperidin, narirutin, and hesperidin, and the content were 243.78 ± 2.69, 5.96 ± 0.06, 12.11 ± 0.12, and 136.04 ± 4.24 mg/g, respectively (9). Since PTFC is regarded as the major active ingredient of *C. maxima (Burm.) Merr*., the analysis of its biological functions is of great significance in clarifying the potential treatment fields. Accordingly, this study aims to systematically review the therapeutic efficiency and possible utilization of pure total flavonoids isolated and purified from the dry and ripe peels of *C. maxima (Burm.) Merr*., which is exhibited in Figure 1.

## Chemistry

Flavonoids are yellow pigments structured by two benzene rings (A and B) connected with a heterocyclic ring containing oxygen (C). The classification of subclasses is dependent on the degree of unsaturation and oxidation of the C ring (10). Flavanone (2,3-dihydro-2-phenylchromen-4-one) refers to the major class of flavonoids with the saturated double bond between positions 2 and 3. Flavanone exists in the forms of glycosides and aglycones. The components of PTFC belong to the former, in which naringin and neohesperidin belong to neohesperidosides, and narirutin and hesperidin belong to rutinosides. In general, the metabolism of glycosides comprises gastrointestinal hydrolysis and liver metabolism. The glucose, xylose, rhamnose, and other glycosyl are hydrolyzed in the gastrointestinal tract to form aglycones. Naringenin and hesperetin have been found as the critical flavanones in aglycone forms (11). Citrus taste properties comprise sweetness, bitterness, and sourness (12). The flavanone-7-O-neohesperidosides partly account for the bitter taste of naringin and neohesperidin, while the tasteless flavanone-7-O-rutinosides account for the non-bitter species, hesperidin and narirutin (13).

## Technology

The extraction and utilization of effective fractions in *Citrus* have attracted wide attention based on the abundant germplasm resources of *Citrus* in southeast Asia. Water extraction is the earliest process used for extraction and exhibits the characteristic of low cost and high safety. Since the decoction should follow the rigorous standards of liquid-material ratio, soaking time, and extraction temperature, water extraction has been replaced by organic solution extraction. The addition of ethanol significantly increases the solubility of *Citrus* peel since most flavonoids have phenolic hydroxyl groups that are soluble and precipitated after acidification (14). Moreover, the leaching capacity of alkaline diluted alcohol is significantly lower than that of alkaline solution, which facilitates purification since it is not easily attached to impurities. To further transfer flavonoids from plants, Xiong et al. (15) used the above methods and obtained 14.65 g PTFC dry powder through soaking, heating, concentrating, separating, purifying, and drying processes. On that basis, the purity of total flavonoids of 88% and the yield of ketone extraction of 2.93% were achieved. After verification, the extraction requirements are confirmed as follows: Mass concentration of ethanol = 70%, solid-liquid ratio = 1:14, decoction time = 60 min and decoction for twice.

Later, Zeng et al. (16) proposed that the single ethanol method combined with ultrasonic-assisted extraction (UAE) and microwave-assisted extraction (MAE) as auxiliary techniques is capable of saving energy and time, and significantly increasing the yield of PTFC, thus revealing that the application of UAE facilitates the extraction process and reduces solvent consumption (17). MAE has also been well accepted for the extraction of bioactive compounds from non-edible parts due to the short time and less solvent consumption and the extracted amount of the above compounds is reported to be comparable to or higher than that of the edible part of the same plant (18, 19). In the future, more well-designed parameter settings are required, and technical research should be conducted to increase efficiency, reduce cost consumption, and protect environmental safety.

## Exploration on biological function of pure flavonoids compounds exerted from *Citrus maxima (Burm.) Merr*.

The following sections summarize the existing main efficacy of PTFC, discussing its treatment on non-alcoholic liver disease, cancer, and intestinal barrier damage and related mechanisms based on studies listed in Table 1.

**TABLE 1:** Summary of biochemical effects exerted by PTFC.

Function	Ingredient	Purity	Source	Experimental dose	Vitro/ Vivo	Object	Biochemical effects
On non-alcoholic fatty liver disease	PTFC	88. 50%	Self-collection	50, 100, and 200 mg⋅kg^–1^	Vivo	Male SD Rats	Active regulation of lipid metabolism (23)
	PTFC	76. 22%	Self-collection	25, 50, and 100 mg⋅kg^–1^	Vivo	Male C57BL/6 mice	By adjusting SIRT1/PGC-1α signaling pathway, enhance liver antioxidant capacity and prevent the occurrence and development of non-alcoholic steatohepatitis (NASH) (26)
	PTFC	76.22%	Self-collection	25, 50, and 100 mg⋅kg^–1^	Vivo	Obesity induced non-alcoholic fatty liver disease (NAFLD) model of rats	Attenuated hepatic lesions with significantly decreased NAFLD activity scores and suppressed both systemic and intrahepatic inflammation (27)
	PTFC	76. 22%	Self-collection	25, 50, and 100 mg⋅kg^–1^	Vivo	Male C57BL/6 mice	Alleviated the inflammatory response of Nash and prevented its progression by regulating the balance of Th17/Treg (28)
On cancer	PTFC	74.04%	Self-collection	0.25–2 mg/mL	Vitro	Kasumi-1, HL-60 and K562 cell line	Inhibits the invasion and migration of human glioblastoma cell via downregulation of MMP-2 and MMP-9 expression and inactivation of p38 signaling pathway (31)
	PTFC	/	Self-collection	0.5–8 μM	Vitro	U937 (CRL-3253), HL60 and K562 cell line	Inhibited leukemic cell proliferation and induce caspase-dependent apoptosis (32)
On intestinal barrier damage	PTFC	76. 22%	Self-collection	/; 100 mg⋅kg^–1^	Vitro/ Vivo	IEC-6 cell line; Male Sprague–Dawley rats	Protected intestinal barrier integrity against NSAID-induced small intestine injury by promoting autophagy via PI3K/AKT signaling pathway (9)
	PTFC	76.22%	Self-collection	50 mg⋅kg^–1^	Vivo	Male C57BL/6J mice	Attenuated HFD-induced NASH symptoms by increasing phylogenetic diversity of microbiota dysbiosis and improving bile acid metabolism (36)

### Non-alcoholic fatty liver disease

The unique metabolism of the liver and its correlation with the gastrointestinal tract take on a critical significance in the human physiological function. A wide variety of factors can lead to chronic liver damage (e.g., alcoholism, viral infections, obesity, diabetes, and dyslipidemia), eventually causing steatohepatitis and liver fibrosis (20). Steatosis characterized by the accumulation of lipid in hepatocytes primarily leads to non-alcoholic fatty liver disease (NAFLD) (21). Both oxidative stress and inflammatory response can be beneficial to the aggravation of NAFLD to non-alcoholic steatohepatitis (NASH) (22). Yang et al. (23) found that PTFC is capable of alleviating steatosis by reducing the body weight, lowing serum total cholesterol (TC), low-density lipoprotein cholesterol (LDL-C) and triglyceride (TG), and actively regulating peroxisome proliferator-activated receptor α (PPAR-α) in hyperlipidemic rats. Also, PTFC is capable of correcting dyslipidemia by regulating superoxide dismutase (SOD) and malondialdehyde (MDA) levels (24, 25), thus inhibiting lipid peroxidation. Jiang et al. found the beneficial effects of PTFC on NASH in two studies. They suggested that PTFC intervention can upregulate SIRTI-mediated deacetylated PGC-1α and SOD and downregulate MDA and 8-hydroxy-2′-deoxyguanosine (8-OHdG), which are biomarkers for lipid peroxidation and free radical level, respectively (26). In addition, they also confirmed that PTFC has hepatoprotective and anti-inflammatory functions by inhibiting phosphorylated NF-κB and MAPKs, as well as the abnormal activation of proinflammatory factors (e.g., IL-1β, IL-6, IL-12, TNF-α, and IFN-γ) (27). Furthermore, PTFC is conductive to maintaining the balance of Th17/Treg expressions and their transcriptional regulators RORγt and Foxp3 (28), which relieves the intrahepatic inflammatory response by limiting the immunopathological reaction and inhibiting the secretion of IL-17 and IL-10 (29).

Above all, existing research has concluded that the potential efficiency of PTFC can arise from the regulatory effect on lipid metabolism, anti-oxidation, and anti-inflammation properties. PTFC can be a curative agent for preventing NAFLDs.

### Cancer

Malignancies are initiated by the destruction of DNA replication and aggravated by immune escape, which further results in distant metastasis. Previous research has demonstrated that dietary intake of flavonoids is beneficial to reduce the risk of malignancies (30). Dai et al. (31) uncovered that PTFC invention dose-dependently and time-dependently inhibits proliferation of neoplastic cells and triggers cell apoptosis by upregulating activated poly adenosine diphosphate-ribose polymerase and downregulating Mcl-1. Overexpression of XIAP is common in carcinomas, usually suggesting an unfavorable response to chemotherapy, increased recurrence rate and shorter overall survival. Wu et al. (32) demonstrated that PTFC at a particular concentration range can downregulate XIAP, and the inhibition promotes apoptosis of cancer cells by activating caspases-3, -7, and -9.

Nearly the whole approaches target to remove or kill the neoplastic cells to control the tumor growth and to minimize the number of metastatic cells. These results revealed that PTFC is beneficial to inhibit cell proliferation and induce apoptosis in malignancies, and not involved in the resistance to cancer cell migration and invasion, which needs to be supplemented by more experimental and clinical evidences.

### Intestinal barrier damage

The gastrointestinal tract plays a critical role in regulating the immune homeostasis since it exhibits the largest interface in the body (33). It is accepted that intestinal barrier is the first line of defense against the entry of pathogenic microorganisms into human intestines. Due to the long-term administration of non-steroidal anti-inflammatory drugs (NSAIDs) for tumor chemoprophylaxis and cardiocerebrovascular diseases, compounds and their metabolites are subjected to a prolonged and repeated exposure of the intestinal mucosa, thereby impairing the intestinal barrier (34). Toxins, bile acids, and proteolytic enzymes all aggravate the intestinal barrier with the increase in intestinal mucosal permeability (35). Chen et al. (9) applied PTFC to rats with NSAID-induced small intestine injury and observed that Atg5, LC3-II, TJ, and the LC3-II/LC3-I ratio are upregulated, while p-PI3K and p-Akt are downregulated, suggesting that PTFC maintains the intestinal barrier integrity by promoting autophagy through the PI3K/AKT signaling pathway. They also claimed that PTFC intervention significantly reduces the content of toxic bile acids (e.g., TDCA, DCA, TCA, and CA), and enriches the phylogenetic diversity of gut microbiota in high-fat diet (HFD)-induced rats (e.g., *Bacteroidaceae* and *Christensenellaceae*) (36). That is to say, PTFC has a promising role in protecting human intestinal barrier, and its major effects include optimization of barrier permeability, positive balance of gut microbiota, and improvement of bile acid metabolism, which are considered to be vital in intestinal immunomodulation.

## Extension of pure flavonoids compounds exerted from *Citrus maxima (Burm.) Merr*. treatment based on its components

**FIGURE 2:**
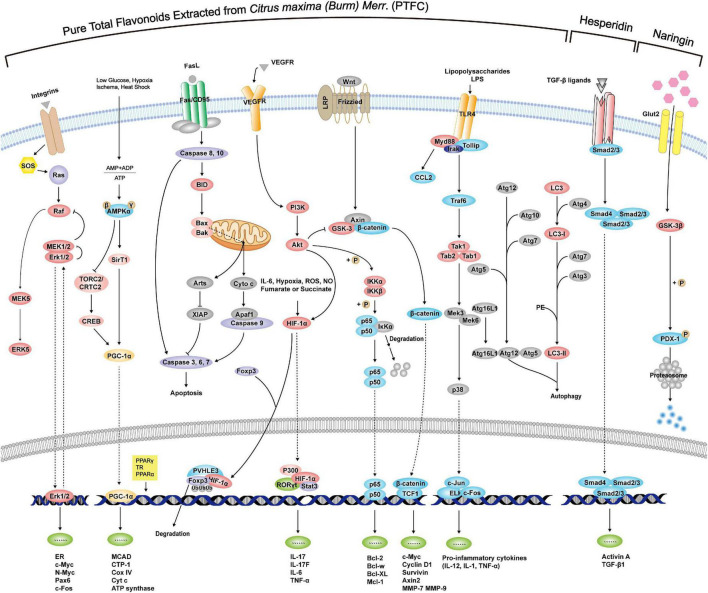
Mechanisms of PTFC and its components through interference with signaling pathways exerting effects of anti-oxidation, anti-inflammation, anti-cancer, and glycolipid metabolism.

According to the above exploration of PTFC treatment, the following sections further excavate the promising effects of its components on liver damage, NAFLD-associated CVDs, malignancies, and intestinal microecological disorder based on studies listed in Table 2, providing new perspectives on the potential functions of PTFC. All the related mechanisms are shown in Figure 2.

**TABLE 2:** Summary of biochemical effects exerted by PTFC components, including naringin, hesperidin, neohesperidin, and narirutin.

Function	Ingredient	Purity	Source	Experimental dose	Vitro/Vivo	Object	Biochemical effects
On liver damage	Naringin	Pure	Solarbio (Beijing, China)	10 μM	Vitro/Vivo	HepG2 cell line and tissue-engineered fatty (TEF) model	Improved lipid metabolism disorders in TEF livers by reducing fatty acid uptake and *de novo* lipogenesis and increasing fatty acid oxidation (38)
	Neohesperid	Pure	Shanghai Yuanye Biological Technology, China	50 mg⋅kg^–1^	Vivo	Male C57BL/6 mice	A good candidate to be dietary supplement for the auxiliary treatment of NAFLD (39)
	Narirutin	75%	Self-collection	150 and 300 mg⋅kg^–1^	Vivo	Male ICR mice	Co-administration with alcohol can alleviate alcohol induced liver damage through preventing lipid formation, protecting antioxidant system and suppressing productions of pro-inflammatory cytokines (40)
	Naringin	/	/	6.25, 12.5, and 25 mg/L	Vivo	Adult wild-type zebrafish and liver-specific EGFP transgenic zebrafish	Contributed to downregulation of mRNA expression of alcoholic injury and lipid metabolism-related genes (41)
	Hesperidin	Pure	Sigma (St. Louis, MO, USA)	200 mg⋅kg^–1^	Vivo	Adult male wistar rats	Co-administered with DEC can against liver fibrosis (42)
On NAFLD-associated cardiovascular diseases	Naringin, Narirutin	Pure	/	25 and 50 μM	Vivo	Male Sprague–Dawley (SD) rats	Prevented Atherosclerotic formation by reducing the expression of CRP, inhibiting the kinases activity of JNK2 and p38, and suppressing the MAPK pathway (47)
	Naringin	/	/	10, 50, 100, and 200 μM	Vitro	HUVECs cell line	Protected endothelial cells from apoptosis and inflammation by regulating the Hippo-YAP pathway (49)
	Hesperidin	/	Florida Department of Citrus (Lake Alfred, FL, USA)	292 mg	Clinical	Healthy, overweight men (50–65 years)	Produced favorable changes in blood pressure (50)
	Hesperidin	Pure	Blue California (Rancho Santa Margarita, CA, USA), The pharmacy at Clinical Center for Atherosclerosis “Tor Vergata” (Rome, Italy)	10 μM; 500 mg/d	Vitro/Clinical	Bovine aortic endothelial cell line, U937 cell line, adults between 21 and 65 years with metabolic syndrome	Stimulated production of nitric oxide in endothelial cells while improving endothelial function and reducing inflammatory markers in patients with metabolic syndrome (51)
	Narirutin	Pure	Sigma–Aldrich (St. Louis, MO, USA)	1–100 μM	Vitro	Arterial rings from Male Sprague–Dawley rats	Vasorelaxing effect and inhibited phosphodiesterase (52)
	Hesperidin	Pure	Xi’an Natural-Field Bio-technique Co., Ltd. (Xian, China)	1–10 μM; 100 and 200 mg⋅kg^–1^d^–1^	Vitro/Vivo	Mouse peritoneal macrophages; Male LDLr^–/–^ mice on C57BL/6JNju	Inhibit atherosclerosis via its pleiotropic effects, including improvement of insulin resistance, amelioration of lipid profiles, inhibition of macrophage foam cell formation, anti-oxidative effect and anti-inflammatory action (60)
	Hesperidin	Pure	/	100 mg⋅kg^–1^d^–1^	Vivo	Male Wistar rodents	Prevented the elevated of oxidative stress, inflammatory markers and liver histology in hyperlipidemia rats (61)
	Naringin	Pure	Shanghai Yuanye Biotechnology Co., Ltd. (Shanghai, China)	100 mg⋅kg^–1^d^–1^	Vivo	Female ApoE^–/–^ mice	Promoted bile acid synthesis by regulating CYP7A1 expression via the gut microbiota-FXR/FGF15 pathway (62)
	Neohesperidin	Pure	Sigma-Aldrich (St. Louis, MO, USA)	1, 10, and 100 μmol/l; 150, 300, and 600 mg	Vitro/Vivo	HepG2 cell line; Homozygous C57BL/6 mice	Exerted lipid-regulating effects via FGF21 and AMPK-SIRT1-PGC-1α signaling axis (63)
	Naringin, Neohesperidin	37 and 47%	Bergavit R^@^ (Bionap, Belpasso, Italy)	150 mg	Clinical	Patients with moderate hypercholesterolemia	Helps reduce the risk of atherosclerosis (64)
	Naringin	Pure	Sigma Chemicals Co. (St. Louis, MO, USA)	100 mg⋅kg^–1^d^–1^	Vivo	Male albino rats	Insulinotropic effects and insulin improving action which in turn may be mediated through enhancing insulin receptor, GLUT4 and adiponectin expression in adipose tissue (68)
	Naringin	95%	HiMedia Laboratories Pvt., Ltd. (Mumbai, India)	100 mg⋅kg^–1^d^–1^	Vivo	Male adult Wistar albino rats	Attenuates β-cell dysfunction through upregulation of PDX-1 (69)
	Naringin	Pure	Sigma Aldrich Chemical Co. (St. Louis, MO, USA)	179.5 ± 10 μM; 100 mg⋅kg^–1^d^–1^	Vitro/Vivo	HepG2 cell line; Male Sprague–Dawley rats	Ameliorates type 2 diabetes mellitus-induced steatohepatitis by inhibiting RAGE/NF-κB mediated mitochondrial apoptosis (70)
	Hesperidin	Pure	/	500 mg	Clinical	Patients with NAFLD	Improved glucose and lipid metabolism, while reduced inflammation and hepatic steatosis (71)
	Hesperidin	Pure	/	25, 50, and 100 mg⋅kg^–1^d^–1^	Vivo	Adult Male albino Wistar rats	Potential antihyperglycemic activity in streptozotocin-induced diabetic rats (72)
	Naringin, Hesperidin	Pure	Sigma Aldrich Chemical Co. (St. Louis, MO, USA)	0.2 g⋅kg^–1^	Vivo	Type 2 diabetic mice	Improves hyperglycemia in type 2 diabetic mice by increasing glucose utilization (73)
	Neohesperidin	98%	Xi’an Xiaocao Biotechnology Co., Ltd. (Xi’an, China)	50 mg⋅kg^–1^ BW	Vivo	Male KK-Ay mice and C57BL/6 mice	Activation of the AMPK pathway and regulation of its target genes, including SCD-1, FAS, and ACOX (74)
On tumor metastasis	Hesperidin	Pure	/	100–600 μM	Vitro	MCF-7 cell line	Combined with chlorogenic acid synergistically inhibited the growth of breast cancer cell via estrogen receptor/mitochondrial pathway (76)
	Hesperidin	Pure	Santa Cruz Biotechnology, Inc. (Dallas, TX, USA)	50, 75, 100, and 125 μg/ml	Vitro	A549 cell line	Induced apoptosis and G0/G1 arrest in A549 cells (77)
	Hesperidin	Pure	/	0, 0.1, 1, and 10 μM	Vitro	A2780 cell line	Inhibited ovarian cancer cell viability through endoplasmic reticulum stress signaling pathways (78)
	Hesperidin	>95%	Yuanye Bio-Technology Co., Ltd. (Shanghai, China)	200 μl	Vivo	4T1 cell line, BALB/c mice	Inhibited metastasis of breast cancer in mice (79)
	Hesperidin	Pure	Santa Cruz Biotechnology, Inc. (Dallas, TX, USA)	25 μg/mL	Vitro	A549, H460, H1975 cell line	Suppressed the migration and invasion of non-small cell lung cancer cells by inhibiting the SDF-1/CXCR-4 pathway (80)
	Hesperidin	Pure	Sigma (St. Louis, MO, USA)	25 mg⋅kg^–1^	Vivo	Albino rats	Upregulated the expression of Smad4 and activin A genes, which has a preventive effect on colorectal cancer (81)
	Hesperidin	∼95%	/	200 mg⋅kg^–1^	Vitro	Female Wistar rats	Reduced the incidence of breast cancer, tumor volume and survival rate and Doxorubicin toxicity (82)
	Hesperidin	Pure	Sigma-Aldrich (St. Louis, Mo., USA)	200 mg⋅kg^–1^	Vivo	Adult male Wistar rats	Decreased the elevation in liver function enzymes, serum AFP level, and oxidative stress markers during the formation of hepatocellular carcinoma via downregulation of the PI3K/AKT pathway (83)
	Hesperidin	Pure	Sigma-Aldrich (St. Louis, Mo., USA)	0.78, 3, 6, 12.5, and 25 mM; 250, 500, and 1,000 ppm	Vitro/Vivo	Human HL60 leukemia cancer cell line; Male Sprague–Dawley rats	Exerted cytotoxic effect on leukemia cancer cell, hypomethylating effect on LINE-1 sequence and ALUM2 repetitive sequences, reduced the diethyl nitrosamine-induced hepatic nodules (84)
	Naringin	Pure	Sigma-Aldrich (St. Louis, MO, USA)	1, 2 mM	Vitro	AGS cancer cell line	Induced lysosomal membrane permeabilization by downregulating mTOR signal and releasing lysosomal cell death protein Cathepsin D lead ERK1/2, p38 MAPKs activation via ROS and BH3-only Bad increase, and Bcl-xL decrease in autophagy mediated cell death in AGS gastric cancer cells (85)
	Naringin	Pure	Sigma Aldrich (USA)	12.5, 25, and 50 μg⋅mL^–1^	Vitro	Human HT-29 cell line	Combined with tunicamycin and BAY 11-7082 induced apoptotic cell death in colon cancer via oxidative stress and the PERK/eIF2α/ATF4/CHOP pathway (86)
	Naringin	98%	Sigma Aldrich (USA)	3, 6, and 10 μM; 60, 120, and 180 mg⋅kg^–1^	Vitro/Vivo	Human malignant glioma U87 cell line; Athymic mice (Crl: CD-1 nuBR)	Inhibited tubulogenesis and reduced cell invasion (87)
	Naringin	98%	Sigma Company (St. Louis, France)	10, 20, and 40 μM	Vitro	Human glioma U251 cell line	Inhibited the invasion and migration of glioblastoma cell via downregulation of MMP-2 and MMP-9 expression and inactivation of p38 signaling pathway (88)
On intestinal microecological disorder	Neohesperidin	Pure	Chengdu Herbpurify Co., Ltd. (Sichuan, China)	50 mg⋅kg^–1^	Vivo	Male C57BL/6J mice	Attenuated weight gain, low-grade inflammation, and insulin resistance in HFD-fed mice via alteration in the diversity and composition of intestinal microbiota, restored gut barrier damage and metabolic endotoxemia (90)
	Naringin	Pure	Shanghai Yuanye Biotechnology Co., Ltd. (Shanghai, China)	100 mg⋅kg^–1^	Vivo	ApoE^–/–^ female mice	Alleviated atherosclerosis by modulating levels of cholesterol and bile acids via gut microbiota remodeling and changes in CYP7A1 and FXR/FGF15 expression (91)
	Naringin, Hesperidin	14.6 ± 0.5%, 72.9 ± 1.6%	Pera-Rio variety oranges (Citrosuco SA, Matão, Brazil)	300 mL/d	Clinical	Healthy female volunteers (20–35 years)	Showed a prebiotic effect, modulating the intestinal microbiota while improving the glycemia and lipid profiles (92)
	Hesperidin	95.50%	Ferrer HealthTech (Murcia, Spain)	100, 200 mg⋅kg^–1^	Vivo	Male Lewis rats	Reinforce prebiotic role via changes in microbiota composition, maintained gut homeostasis by increasing intestinal IgA at a lower dose (93)
	Hesperidin	93%	NUTRAFUR S.A. (Murcia, Spain)	40 and 100 mg⋅kg^–1^	Vivo	Male Sprague–Dawley rats	Ameliorated lipidomic profile, blood pressure, insulin sensitivity, decreased markers of arterial stiffness and inflammation, which positively correlated with Bacteroidaceae family (94)
	Neohesperidin	Pure	Sigma-Aldrich (St. Louis, Mo., USA)	25, 50, and 100 μM; 50 and 100 mg⋅kg^–1^	Vitro/Vivo	HCT116, SW480 cell line; C57BL/6J-APC ^min/+^ mice	Inhibited colorectal tumorigenesis by alterations of gut microbiota, including inducing apoptosis and inhibiting angiogenesis (95)
	Hesperidin	Pure	Self-collection	0.67–4.76 mg⋅mL^–1^	Vitro	*Staphylococcus aureus*, *Bacillus cereus*, *Pseudomonas aeruginosa*, and *Escherichia coli*	Synergize and add the preservative activity of NaNO2 (96)
	Naringin	Pure	Sigma-Aldrich (Bengaluru, India)	370, 390, 410, 430, 450, and 470 μg⋅mL^–1^	Vitro	*P. aeruginosa* MTCC 2488 biofilm	Potentiate the efficacy of ciprofloxacin and tetracycline on *P. aeruginosa* biofilm (97)

### Liver damage

Studies have shown that the prevalence of a HFD, a sedentary lifestyle and alcoholic abuse are the risk factors for liver damage (37). The therapeutic effects of PTFC on liver metabolism affected by overnutrition has been achieved. Furthermore, it is found that naringin could reduce TG and very low-density lipoprotein (VLDL) in the fatty liver model by activating PPAR-α (38), and neohesperidin could facilitate PGC-1α-mediated mitochondrial biogenesis, thus alleviating hepatic steatosis in mice fed with a HFD (39), which further highlights the main contributor in PTFC efficacy.

On the other hand, the effects of narirutin, naringin, and hesperidin on alcoholic liver diseases has also been reported, providing new references for expanding the therapeutic field of PTFC treatment. In mice with a long-term consumption of alcohol, narirutin intake can reduce the serum transaminase, pro-inflammatory cytokines, TG, and TC (40). Zhou et al. confirmed that naringin protects against alcohol-induced liver damage by reducing lipid accumulation and oxidative stress (41). The combination of hesperidin with an anti-inflammatory drug synergistically inhibits alcoholic liver fibrosis (42). As a result, the treatment of PTFC components prevents steatosis caused by chronic alcohol ingestion, thus protecting alcoholic liver diseases. It is therefore suggested that PTFC alleviates alcoholic-induced liver diseases by preventing excessive lipid formation and protecting the antioxidant system, which should be validated in depth through animal studies and clinical trials.

### Non-alcoholic fatty liver disease-associated cardiovascular diseases

Non-alcoholic fatty liver disease increases the risk of CVD, and it has been found as the most common cause of death of non-alcoholic liver diseases (43). Myocardial infarction, stroke, and revascularization are common risks of CVD, which are significantly correlated with NAFLD (44). However, a *post hoc* analysis indicated that the hazard risk of NAFLD population with body mass index ≥23 kg/m^2^ for incident CVD is lower than those non-obese population (45), thus suggesting that myocardial infarction or stroke-induced vascular dysfunction is likely to be the pivotal element for CVD rather than obesity. Atherosclerosis in NASH patients is usually confirmed through the measurement of calcification and aortic stiffness (46). Naringin and narirutin are found to block the formation of scar tissue and vascular stenosis in atherosclerosis cases by downregulating pro-inflammatory factors (47). Similarly, naringin is able to prevent endothelial dysfunction with a decrease in the expressions of inflammatory cytokines (IL-6, TNF-α, and IL-1β) via Nox4/NF-κB, PI3K/AKT and Hippo-YAP pathways, thus relieving atherosclerosis (48, 49). Existing research has found that hesperidin intervention leads to favorable changes in endothelium-dependent microvascular reactivity by decreasing the concentrations of circulating inflammatory biomarkers (e.g., high-sensitivity C-reactive protein, serum amyloid A protein, and soluble E-selectin) and regulating nitric oxide bioactivity (50, 51). Narirutin relaxes the vascular system by stimulating the phosphorylation of endothelial nitric oxide synthase and voltage-dependent potassium current in arterial smooth muscle cells (52). Accordingly, the effects of PTFC compounds on NAFLD-associated CVD are primarily attributed to the inhibition of systematic inflammation. The persistent inflammatory responses trigger the aggravation of hepatic steatosis, NASH to liver fibrosis, thus significantly increasing the risk of CVD. An early intervention for NASH may reduce the incidence of cardiovascular events. Liver biopsy has been employed as the gold standard for the diagnosis of NASH though it is not highly acceptable due to the invasive examination and high medical costs. Although emerging blood makers and non-invasive imaging methods have been applied to the diagnosis of fibrosis and cirrhosis (53, 54), steatosis identification and inflammation evaluation should be conducted to make the progression of NASH. There have been rare typical manifestations of early stage NASH, so it is urgent to search for a reliable and accurate diagnostic method.

Interestingly, abnormal lipid metabolism and its transportation trigger the development of atherosclerosis (55, 56). NAFLD-induced excessive lipid uptake results in the abundant synthesis of hepatic TG and overproduction of VLDL particles assisting the mobilization of liver fat to peripheral tissues (57, 58). The accumulation of VLDL and TG on the arterial wall, alongside inflammation response and oxidative stress, leads to a heavy burden of atherosclerotic plaques (59). Free fatty acids (FFAs) are the product of lipolysis in the portal circulation. Sun et al. (60) built a lipoprotein receptor deficient model in atherosclerosis mice fed with HFD and discovered that hesperidin can suppress the formation of atherosclerotic plaque and macrophage foam cells by inhibiting the activities of enzymes involved in hepatic FFAs and TG synthesis, thus further suppressing the cholesterol transportation from peripheral tissues to the liver and then preventing hepatic steatosis. Also, hesperidin could maintain the redox homeostasis and prevent lipidemic stress in hyperlipidemia rats (61). Wang et al. (62) suggested that naringin more significantly mitigates atherosclerosis than hesperidin (55.92% vs. 42.87%). Their data revealed that the concentration of naringin in the gastrointestinal tract is higher than that of hesperidin, and a small amount of naringin in the liver is subsequently absorbed. Naringin facilitates the transformation from cholesterol to bile acids and promots their excretion from the liver by regulating the gut microbiota structure. Moreover, some naringin absorbed in the liver facilitates the reverse transport of cholesterol (62). Neohesperidin consistently exerts a potent hypolipidemic effect on HepG2 cells enriched in FFAs and reverses lipid-related pathological changes in the acute or chronic dyslipidemia mouse model (63). A kind of citrus juice containing neohesperidin and naringin significantly alleviates subclinical atherosclerosis by reducing lipoprotein content and carotid intima-media thickness within 6 months (64).

Increasing data have indicated that obesity is not the necessary factor for cardiovascular events in NAFLD population, but boosts the development of atherosclerotic plaques to a certain extent. Notably, NAFLD population with dyslipidemia is highly risky for cardiovascular events. The ingredients of PTFC, especially hesperidin and naringin, possess therapeutic efficiency on lipid deposition and adiposity, which dramatically inhibit the formation of atherosclerosis via blocking the secretion and transport of redundant lipid.

Glucose abnormality induced by insulin resistance and beta cell dysfunction is a hallmark of NAFLD, which takes on a great significance in the pathogenesis of CVD (65, 66). Elevated FFAs released by enlarged adipose mass remarkably restrict the anti-lipid effect of insulin and even lead to insulin resistance, which in turn aggravates dyslipidemia and pathoglycemia (67). Naringin exerts the insulinotropic effect by increasing the expression levels of insulin receptors and adiponectin in adipose tissues (68). Also, naringin attenuates mellitus-mediated steatohepatitis by upregulating the transcription factor PDX-1 that regulates insulin secretion and maintains β-cells mass (69), and inhibiting hyperglycemia-mediated oxidative stress and pro-inflammatory cytokine secretion (70). Yari et al. (71) suggested that hesperidin supplementation controls fatty liver indexes by inhibiting inflammation, while enhancing insulin sensitivity and fasting glucose. Meanwhile, hesperidin and naringin contribute to glucose uptake by enhancing hepatic glycolysis and glycogen content, and lowering hepatic gluconeogenesis (72, 73). Neohesperidin suppresses fat accumulation and reduces the size of adipocytes, which further improves oral glucose tolerance and insulin sensitivity (74).

A conclusion is drawn that, PTFC, at least in part, plays a positive role in the treatment of CVDs by suppressing vascular senescence, improving lipid profile and maintaining glucose homeostasis for its active ingredients. Moreover, their activities of controlling persistent inflammatory response and oxidative stress facilitate the prevention of CVD. However, inconsistent results on glucolipid metabolism have been obtained in some randomized controlled trials (RCTs). Motallaei et al. (75) reported that the meta-analysis on the intake of orange beverage associated with relieving serum TC and insulin resistance is poorly qualified, which fails to show a significant effect of citrus drinks on cardiometabolic risk factors. The above phenomenon can be explained by the poor bioavailability of flavonoids in the human body. Since the absorption of flavonoids focuses on the colon, individualized differences in gut microbiota compositions and activities result in varied therapeutic efficiency. Therefore, in addition to early intervention of steatosis to prevent CVD, it is also essential to mediate the intestinal microecology, which can facilitate the absorption of PTFC.

### Tumor metastasis

The growth of the primary tumors often do not pose major health threats except for those growing in sensitive and restrictive organs, such as the brain. Transformation into a cell capable of metastasis, acquiring the capabilities to escape the primary tumor, to enter vascular systems, to invade, and to colonize secondary organs, is more of a concern. Hesperidin is reported to control tumor growth by regulating mitochondria production (76), arresting cell cycle progression (77), and inhibiting cell viability via the endoplasmic reticulum stress signaling pathway (78). It also can inhibit the metastatic potential by suppressing metastases growth *in vivo* and cell migration and invasion *in vitro* (79, 80). Existing research indicated that hesperidin contributes to chemotherapy by modulating Smad4 and the activin A signaling in colon cancer (81), and downregulating Ki-67 expression in breast cancer (82). During the process of diethyl nitrosamine-induced hepatocellular carcinoma, the addition of hesperidin preserves liver tissue integrity, improves liver function (83), and exerts a hypomethylating effect (84). As the major component in PTFC, naringin is considered an anti-tumor agent and adjuvant in the combination therapy by inducing lysosomal permeabilization and autophagy in gastric cancer (85). It induces mitochondrial and cellular apoptosis in colon cancer via inhibiting NF-κB and endoplasmic reticulum stress (86). Though downregulating MMP-2 and MMP-9 and inactivating the p38 signaling, naringin inhibits angiogenesis and cell invasion in glioblastoma cancer (87, 88).

Due to the anti-tumor capacities of hesperidin and naringin in inhibiting metastatic potential, blocking neovascularization, and strengthening chemical protection, the anti-tumor function of PTFC appears to be consistent and reliable. Considering some uncertain aspects of cancer treatment, more *in vivo* and *in vitro* studies are needed to validate the anti-tumor effect of PTFC.

### Intestinal microecological disorder

Besides the physical barrier created by intestinal epithelial cells, the symbiotic relationship between intestinal microbiota and host also contributes to the intestinal immunity. The gut microbiota complements human genome functions based on its wide range of metabolic properties. However, the individualized difference in gut microbiota changes significantly due to diet, antibiotic use, and lifestyle (89).

It is found that neohesperidin administration changes the structure of gut microbiota by decreasing the intestinal ratio of *Firmicutes* to *Bacteroidetes* and enhances gut barrier integrity by mitigating serum metabolic endotoxemia in obese mice, which is reversed by the antibiotic treatment. Increasing evidence suggested that the profitable effect of neohesperidin on the obese population is largely dependent on gut microbiota (90). Naringin alleviates atherosclerosis by modulating the abundances of *Bifidobacterium*, *Bacteroidetes*, *Clostridium*, and *Eubacterium* (91). Fidélix et al. (92) revealed that the absorption and metabolism of hesperidin and naringin increase the abundance of probiotics, thus ameliorating the glycemia and lipid profiles. The immunomodulatory effect of hesperidin on the gut-associated lymphoid tissue is achieved by increasing the proportions of *Lactobacillus* and *Bifidobacterium* (93). Furthermore, an increased dose of hesperidin supplementation reduces the risk of CVD by modulating the metabolism of the *Bacteroidaceae* family (94). Feeding with fecal of neohesperidin-treated mice yielded a considerable inhibition of colon cancer, suggesting that the adjustment of neohesperidin on gut microbiota may be a promising strategy for cancer (95).

Notably, knowledge of PTFC compounds as antimicrobial agent is equally attractive. The current research indicates that hesperidin combining with widely used NaNO2 has synergistic antibacterial activity against *Bacillus cereus*, *Staphylococcus aureus*, *Escherichia coli*, and *Pseudomonas aeruginosa* (96), showing the advantages of nutrients as safe natural bio-preservatives to reduce the hazards of overuse of chemical preservatives. Ciprofloxacin and tetracycline are antibiotics for *P. aeruginosa*, and the addition of naringin potentiates their efficacies to further manage bacterial infection (97). As for methicillin-resistant *S. aureus* that has developed resistance against most of the antibiotics and resulted in life-threatening outbreaks, naringin, hesperidin and neohesperidin are all strongly supported to be the adjuvant antimicrobial agent via the docking interactions (98).

Overall, the regulatory effect of its components on raising the proportion of beneficial bacteria implies the improvement of PTFC on chronic diseases may begin from the impact of its components on gut microbiota, and protecting intestinal microecological homeostasis can be the priority of human health. Based on the efforts on bacteriostasis by its components, the positive effect of PTFC on intestinal immunity has been affirmed again. Regional factors and personal habits lead to individualized differences in the dynamic equilibrium of intestinal microecology, which may be eliminated by the long-term administration of PTFC through regulating the structure of intestinal microbiota or clearing relevant pathological factors.

## Bioavailability

Most flavonoids are extensively absorbed in the intestine and then transported to the liver for the further metabolization. The metabolites formed in the liver can re-enter enterohepatic circulation through hydrolysis of bile excretion to aglycones via gut microbiota or being directly excreted in urine or feces (99). However, flavonoids are poorly absorbed through the gastrointestinal tract (100), and great efforts have been made on enhancing the bioavailability of flavonoids by inhibiting relevant enzymes, altering food intakes, and increasing dissolution rate (101–103). Xia et al. (104) demonstrated that neohesperidin modified with an immobilized lipase (40 mg/ml catalyst, 50°C of reaction temperature and 18 h of reaction time) can enhance the lipophilicity, thus improving the applicability in lipophilic media and enhance bioavailability *in vivo*.

Additionally, the low activity of α-rhamnosidase serves as a limiting step for hesperidin degraded by colonic microbiota (105, 106). Pereira-Caro et al. (107) reported that *Lactobacillus rhamnosus* exhibits the rhamnosidase activity, which is cooperated with *Bifidobacterium* in the *in vivo* colonic catabolism of hesperidin. They also highlighted that the chronic intake of *Bifidobacterium* is significantly beneficial to the enhancement of hesperidin bioavailability (108), suggesting that the long-term intake of PTFC assists the expansion of probiotic communities, also in turn promotes the absorption of PTFC. Unlike the large difference of hesperidin content in PTFC, plasma concentration of narirutin is significantly lower than that of hesperidin (109). The α-rhamnosidase serves as the catalyst to boost the absorption of naringin via the conversion from rutinoside to glucoside (110). Accordingly, α-rhamnosidase activity is still the critical issue to limit bioavailability. The commensal intestinal microecology and its substantial gene pool has been validated to significantly regulate the bioavailability and metabolism of nutrients (111). In addition to increasing the activity of α-rhamnosidase, microbiome profiling combined with powerful machine learning algorithms can be employed to select more appropriate biomarkers for flavonoid metabolism from microorganisms, thus enhancing the clinical response and efficacy to PTFC.

A clinical study suggested that the solubility of hesperidin in juice is a vital factor for bioavailability since its excretion and maximal plasma concentration are correlated with the soluble hesperidin concentration in juice, instead of the total hesperidin intake (6, 112). As a result, encapsuling hesperidin by the nanotechnology is confirmed as a promising strategy to enhance the bioavailability of hesperidin. Also, a reduced particle size facilitates the interaction with intestinal cells and gut microbiota, thus weakening the demand of α-rhamnosidase hydrolysis (113). Meanwhile, the nanoparticulate systems were employed for the naringin formulations to prevent drug cleavage in the lumen or the gut under harsh pH and enzymatic conditions of gastrointestinal tract, providing a sustained delivery of naringin (114).

That is to say, nanotechnology is capable of increasing the encounter area and reducing gastric lysis, which can be extensively used to modulate the release and absorption of bioactive fractions. What’s more, its physicochemical properties should be further modified to enhance the solubility and permeability with the use of nanotechnology. As a result, efforts should be made to assess the effects of nanotechnology on the metabolism, bioavailability, and efficacy of PTFC.

According to studies on the comparison of various *Citrus* fruits (115), the difference of efficacy lies in the total flavonoids content and the main component object. It is reported that the kind of *C. maxima (Burm.) Merr.* has the relatively stronger anti-inflammation, anti-oxidant and bactericidal effects due to it contains a large amount of naringin and hesperidin (116, 117). The total flavonoids content is found to increase with maturity stages (118), so standard planning for the shape, weight and skin luster of *C. maxima (Burm.) Merr.* before picking may be the new focus associated with the absorption of nutrients.

## Conclusion

In brief, the effects of lipid-lowering, anti-inflammation, anti-oxidation, anti-cancer, anti-bacterial, and intestinal barrier protection of PTFC were reviewed and novel prospects were put forward in accordance with the findings on its components. Besides, paying attention to the effects of PTFC on gut microbiota may contribute to the enhancement of therapeutic efficacy, while well-designed experiments and clinical trials are still required to further clarify the specific application of PTFC in clinical practices. Meanwhile, it is found that the mature stage of raw material affects the total flavonoids content, the manufacturing methods affects PTFC purity, and delivery system and individual intestinal microecology affect the specific bioavailability. Thus, optimization on fruit cultivation system and picking standard, and more elaborate investigations on facilitating their controlled release and actual potency in blood should be further conducted to address the poor bioavailability, as well as effective productive and easier methods to separate and extract PTFC should be explored to improve purity.

To the best of our knowledge, this study has summarized the extraction technology, chemical properties, and biological effects of PTFC, which may boost the development of their biological profiles in human disease treatment. Of course, studies on pharmacokinetic parameters, toxicity testing, effective dose assessment, and adverse reaction are of significance before PTFC is officially used as a clinical therapeutic agent.

## Author contributions

SD and PW conducted the data disposal. XP, LZ, and LQ wrote the manuscript. XJ and WC participated in the discussion. SD, PW, SR, and LS revised and edited the manuscript. All authors have read and agreed to the published version of the manuscript.

## Abbreviations

PTFC: pure total flavonoids extracted from *Citrus maxima (Burm.) Merr.*
TCM: traditional Chinese medicine
HPLC: high-performance liquid chromatography
UAE: ultrasonic-assisted extraction
MAE: microwave-assisted extraction
NAFLD: non-alcoholic fatty liver disease
NASH: non-alcoholic steatohepatitis
TC: total cholesterol
LDL-C: low-density lipoprotein cholesterol
TG: triglyceride
PPAR-α: peroxisome proliferator-activated receptor α
SOD: superoxide dismutase
MDA: malondialdehyde
8-OHdG: 8-hydroxy-2′-deoxyguanosine
VLDL: very low-density lipoprotein
HFD: high-fat diet
VEGF-C: vascular endothelial growth factor-C
CVD: cardiovascular disease
FFAs: free fatty acids
NSAIDs: non-steroidal anti-inflammatory drugs.
